# Protocol for predicting drug-resistant protein mutations to an ERK2 inhibitor using RESISTOR

**DOI:** 10.1016/j.xpro.2023.102170

**Published:** 2023-04-27

**Authors:** Nathan Guerin, Teresa Kaserer, Bruce R. Donald

**Affiliations:** 1 Department of Computer Science, Duke University, Durham, NC 27708, USA; 2 Institute of Pharmacy/Pharmaceutical Chemistry, University of Innsbruck, 6020 Innsbruck Austria; 3 Department of Biochemistry, Duke University Medical Center, Durham, NC 22710, USA; 4 Department of Chemistry, Duke University, Durham, NC 27708, USA; 5 Department of Mathematics, Duke University, Durham, NC 27708, USA

**Keywords:** Bioinformatics, Cancer, High-throughput Screening, Protein Biochemistry, Structural Biology, Biotechnology and Bioengineering, Computer Sciences

## Abstract

Prospective predictions of drug-resistant protein mutants could improve the design of therapeutics less prone to resistance. Here, we describe RESISTOR, an algorithm that uses structure- and sequence-based criteria to predict resistance mutations. We demonstrate the process of using RESISTOR to predict ERK2 mutants likely to arise in melanoma ablating the efficacy of the ERK1/2 inhibitor SCH779284. RESISTOR is included in the free and open-source computational protein design software OSPREY.

For complete details on the use and execution of this protocol, please refer to Guerin et al..^1^

## Highlights

• RESISTOR is an OSPREY-based algorithm for predicting resistance mutations
• RESISTOR employs structure and sequence data to predict resistance mutations
• RESISTOR uses Pareto optimization to provide ranks of putative resistance mutations
• RESISTOR and OSPREY are free and open-source software programs

## Abstract (editor highlights)

Publisher’s note: Undertaking any experimental protocol requires adherence to local institutional guidelines for laboratory safety and ethics.

## Graphical abstract

**Figure:**
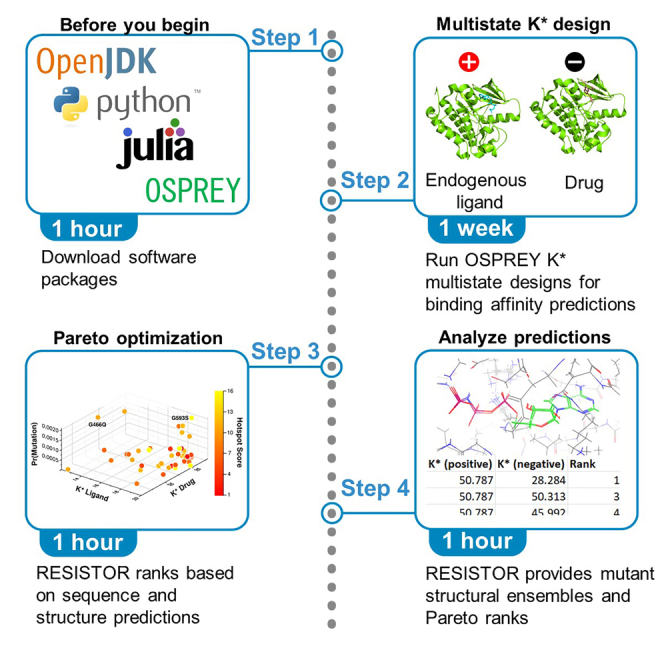

## Before you begin

**Timing: 1 h**

This section describes the minimal hardware and operating system requirements, where to obtain the requisite software and its installation procedure, and the file formats of the sequence and structural inputs required to run RESISTOR. For the purposes of demonstration, we use RESISTOR to predict resistance mutations on the ERK2 kinase to the inhibitor SCH772984 (hereafter referred to as SCH7). Previously, we have used RESISTOR to prospectively predict resistance mutations in EGFR and BRAF, which we then validated experimentally.^1^ In addition, we have employed aspects of RESISTOR, including multistate K∗ design and mutational signature probabilities, in other applications, such as our development of algorithms like BBK∗ (Branch and Bound Over K∗)^2^ and our predictions of resistance-conferring mutations to inhibitors of kinases such as KIT, EGFR, ABL1, and ALK.^3^

Here we offer abbreviated definitions and references for the terminology we use throughout this protocol. *Positive design* is the use of computational protein design algorithms to improve an objective, such as ligand binding. *Negative design* is the opposite, i.e., the goal is to make an objective worse, such as to ablate binding. RESISTOR uses *multistate design*,^4^^,^^5^^,^^6^^,^^7^^,^^8^ or both positive and negative design in parallel, to mimic how mutations affect the competitive balance between a protein’s endogenous ligand and a competitive inhibitor. Resistance can occur via a protein’s increased activity with its endogenous ligand, decreased binding with an inhibitor, or a combination of these factors.^1^^,^^3^^,^^6^^,^^7^

RESISTOR employs Pareto optimization over positive and negative design, mutational signature probabilities, and hotspot scores to rank prospective resistance mutants. The positive and negative design portions use the *K∗ algorithm*^9^ implemented in OSPREY,^10^ which generates low-energy molecular ensembles to compute the partition functions the algorithm uses to provably approximate binding affinity, $K_{a}$.^4^^,^^9^ *Mutational signature probabilities* are derived from data provided by Alexandrov et al.^11^ and denote the probability a DNA base will mutate to another base in a given sequence context and cancer type. A *hotspot score* is the number of sequences with a mutation at a particular residue location which multistate design criteria predicts as structural resistance mutations.^1^^,^^3^

**Figure 1:**
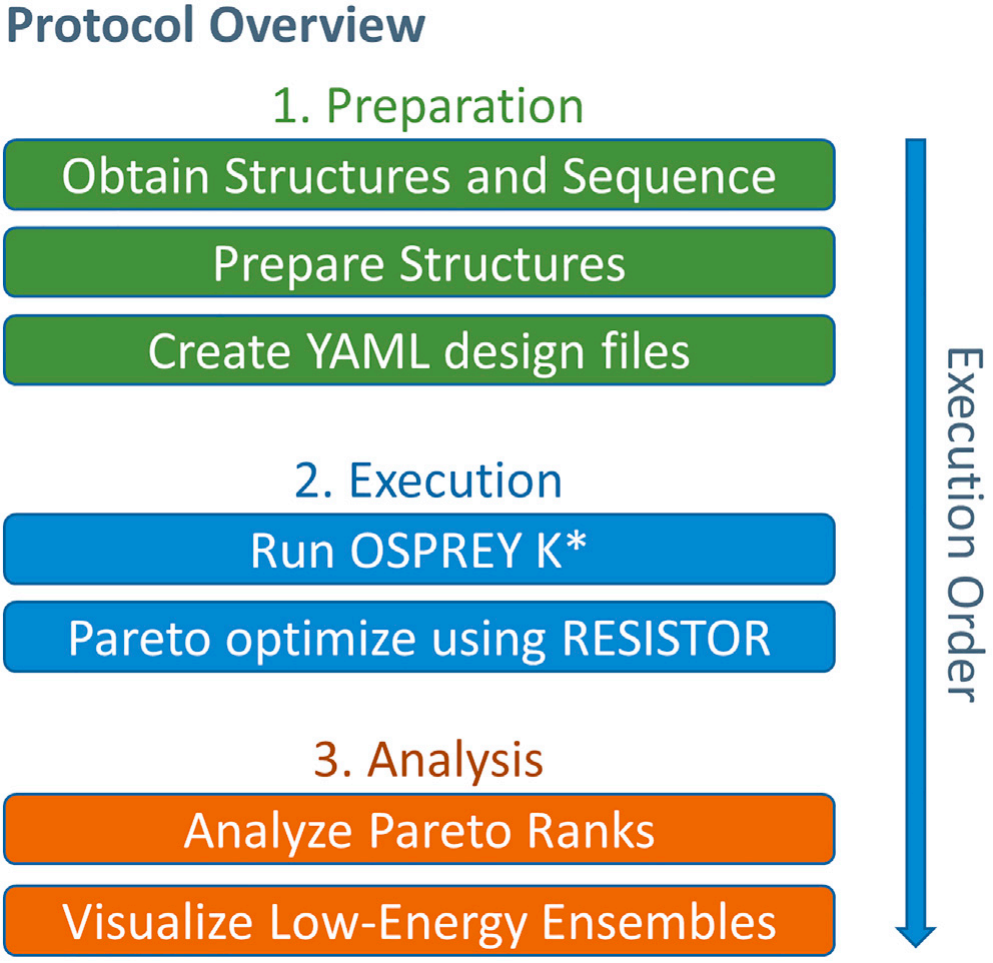
Conceptual overview of the main steps involved in executing the RESISTOR protocol The first phase, Preparation, involves obtaining the positive and negative design structure files in PDB format, along with the corresponding cDNA sequence. The structures need to be prepared for OSPREY K∗ design. Finally, the structures and additional inputs (outlined below) must be collected into a YAML design file. The second phase, Execution, involves using the OSPREY K∗ algorithm to compute provable approximations to the binding constant, $K_{a}$, and using RESISTOR to filter the results, assign mutational probabilities, and Pareto optimize. The final phase, Analysis, is where the user examines the RESISTOR-provided output of Pareto ranks and low-energy molecular ensembles. The details involved in each of these steps are explained comprehensively in this STAR Protocol.

Figure 1 contains a conceptual overview of the RESISTOR protocol’s main phases.

### Hardware and software

RESISTOR requires a minimum of 32 GiB of RAM and 5 GiB of free hard disk space. You also will need to have a good text editor on your computer: vim, emacs, or any other text editor that can be used for editing ASCII characters will do; programs like Microsoft Word or LibreOffice Writer will not. Our demonstration of the protocol is on a Linux operating system, although with minor adjustments the process below could be carried out on Windows and macOS operating systems.

### Installing the Software Dependencies

**Timing: 20 min**

RESISTOR requires Java 17, Miniconda, AmberTools, Julia, and OSPREY.

1. Install Java 17
  a. Download an archive for the latest version of Java 17 for your platform from https://jdk.java.net/archive/
  b. Extract the archive to a location on your computer, e.g., $HOME/java/jdk-17.0.2.
  c. In your shell’s profile, set the JAVA_HOME environment variable to the location you extracted the archive to, and add the java executable to your path, e.g.,
    export JAVA_HOME=$HOME/java/jdk-17.0.2
    export PATH=$PATH:$JAVA_HOME/bin
  d. Verify java is available on your shell’s path by opening a new terminal window, typing java -version, and hitting enter. You should see output like the following:
    > java -version
    openjdk version "17.0.2" 2022-01-18
    OpenJDK Runtime Environment (build 17.0.2+8-86)
    OpenJDK 64-Bit Server VM (build 17.0.2+8-86, mixed mode, sharing)
    Video S1 demonstrates this procedure on Fedora Linux.

2. Install Python using Miniconda
  a. Download the latest Python 3 version of Miniconda from https://docs.conda.io/en/latest/miniconda.html.
  b. Run the interactive installer, e.g.,:
    > sh Miniconda3-latest-Linux-x86_64.sh
  c. When the installer asks you “Do you wish the installer to initialize Miniconda3
    *by running conda init? [yes|no]*”, type yes and hit enter.
  d. Close and re-open your shell.
  e. Now, when you log into your shell, a Miniconda environment is activated. Run the following command so its environment is *not* activated by default.
    > conda config --set auto_activate_base false
    Video S2 demonstrates these steps on Fedora Linux.

3. Install AmberTools
  a. Install AmberTools22 using the installation instructions for “Binary distribution via Conda” on https://ambermd.org/GetAmber.php#ambertools. In short:
    > conda create --name AmberTools22
    > conda activate AmberTools22
    (AmberTools22)> conda install -c conda-forge ambertools=22 compilers
  b. To verify that AmberTools is correctly installed, type:
    > antechamber -h
    A help message for the antechamber program should be displayed.
    Video S3 demonstrates these steps on Fedora Linux.
    ***Optional:*** You can install the conda-packaged yamllint program into this conda environment. Yamllint is used to check the syntactic validity of YAML documents:
    > conda activate AmberTools22
    (AmberTools22)> conda install -c conda-forge yamllint
    If you choose to install yamllint, you should also create a default configuration file that disables its line length check. To do so, create the file $HOME/.config/yamllint/config (and the intermediary directories as necessary), and add the following content:
    extends: default
    rules:
     line-length: disable

4. Install Julia
  a. Download and extract the latest stable release of Julia. RESISTOR was developed using Julia v1.6, but any v1 release of Julia post Julia 1.6 should work.
    i. Go to https://julialang.org/downloads/ to get the latest Julia package.
    ii. Download the architecture-specific Linux archive to your computer.
    iii. Extract the archive to a location on your computer, e.g., $HOME/julia/julia1.8
  b. In your shell’s profile, add the executable to your path, e.g.,
    export JULIA_HOME=$HOME/julia/julia1.8
    export PATH=$PATH:$JULIA_HOME/bin
  c. Close and re-open your shell. Then, to verify that Julia is correctly installed, type:
    > julia --version
    which should print out the version of Julia you downloaded.
    Video S4 demonstrates these steps on Fedora Linux.

5. Install OSPREY with RESISTOR
  a. Download OSPREY version 3.3 from https://github.com/donaldlab/OSPREY3/releases/3.3-resistor
  b. Extract the OSPREY distribution:
    > tar --file osprey-3.3.tar --extract
  c. Add the OSPREY executable to your PATH for simplified access. Assuming you have extracted the archive in the previous step in your home directory, add the following line to your shell’s profile file:
    export OSPREY_HOME=$HOME/osprey-3.3
    export PATH=$PATH:$OSPREY_HOME/bin
  d. Verify you have OSPREY on your path by executing the following command in the terminal, which should display help text:
    > osprey affinity --help
    Video S5 demonstrates this procedure on Fedora Linux. If you do not see the help text, see troubleshooting 1.

Video S1. Demonstration of downloading and installing Java 17, related to installing the software dependencies step 1

Video S2. Demonstration of downloading and installing Miniconda, related to installing the software dependencies step 2

Video S3. Demonstration of installing AmberTools into a conda environment, related to installing the software dependencies step 3

Video S4. Demonstration of downloading and installing Julia, related to installing the software dependencies step 4

Video S5. Demonstration of downloading and installing OSPREY, related to installing the software dependencies step 5

### Obtaining the sequence and structure files

**Timing: <1 h**

6. Download your positive and negative design structure files
  a. Navigate to the Protein Data Bank (https://www.rcsb.org/) in your browser.
  b. Search for the protein of interest. You will need to download a structure of the protein bound to the drug and the protein interacting with the endogenous ligand.
  c. For ERK2 bound to SCH7, search the Protein Data Bank for PDB ID 4qta^12^ and download the file in PDB format.
  d. For ERK2 bound to AMP-PNP (adenylyl-imidodiphosphate, an analog of ATP), search the Protein Data Bank by PDB ID 2y9q^13^ and download the file in PDB format.

7. Download the coding DNA sequence.
  ***Note:*** There are many places on the internet to download DNA sequences. For sequences of proteins implicated in carcinogenesis, such as ERK2, the COSMIC database^14^ is one such good choice.
  a. In a web browser, navigate to https://cancer.sanger.ac.uk/cosmic.
  b. Search for ERK2 and go to the gene view.
  c. Download the cDNA sequence (ENST00000215832.10) in FASTA file format.

8. Choose your cancer-type specific mutational probabilities JSON file.
  a. Identify the probabilities file you need. For this protocol, we will use the melanoma probabilities file, *melanoma.json.*
  b. Mark down the path to this file, which you will use in Assigning Pareto Ranks step 11.
    ***Note:*** The RESISTOR directory within the OSPREY distribution (osprey-3.3/resistor) contains mutational probability files for melanoma, non-small cell lung cancer, stomach cancer and pancreatic cancer. It is also possible to create your own mutational probabilities file, which is covered elsewhere.^15^
    **CRITICAL:** Ensuring that the following prerequisites are met helps avoid downstream prediction problems: 1. When possible, use high-quality, high-resolution structures. While the cut-off for resolution is still a matter of discussion in the scientific community, previous successful designs have used X-Ray diffraction resolutions ranging between 1.4 and 3.15 Å.^6^^,^^7^^,^^16^^,^^17^^,^^18^ We have also had success with cryo-EM resolutions between 3.4 and 11.5 Å. For NMR structures, we recommend that the structure determination use RDCs; 2. Check that the residue numbers and amino acid types in the positive and negative protein structures are the same, e.g., ALA 10 in the structure for the positive design and ALA 10 in the structure for the negative design refer to the same residue; and 3. that the cDNA sequence translates to the amino sequence in the structure files, i.e., they represent the same genetic variant. Furthermore, the FASTA file must begin with the codon that translates to residue number 1. In this example, PDB ID 4qta (ERK2:SCH7) has a resolution of 1.45 Å, PDB ID 2y9q (ERK2:AMP-PNP) has a resolution of 1.55 Å, and the residue numbering in the two structures are the same and correspond to the canonical numbering also used in the FASTA file. See Video S6 for a demonstration of carrying out these checks.
    If your checks indicate discrepancies exist, you will need to manipulate the files to resolve them. As the PDB and FASTA file formats are standard in the fields of structural biology and bioinformatics there are many tools available for their manipulation, including Maestro^19^ for manipulating structural information. Yet as both file formats are defined in human-readable ASCII text, oftentimes the simplest way to make any necessary tweaks in the files is with a standard text editor, such as emacs or vim.
    In cases where empirical structures are not available, it is possible to use docking, homology modeling, or other computational modeling techniques to generate structures.^1^^,^^6^^,^^7^^,^^17^^,^^20^ For example, computational tools such as Modeller^21^ or Alphafold^22^ could be used to predict an initial protein structure, and docking tools such as AutoDock Vina^23^ or those included in Maestro^19^ could be used to dock the positive and negative design ligands.^1^^,^^3^ With the recent explosion of available structural models, such as the Alphafold Protein Structure Database,^24^ it may even be the case that a computationally predicted starting structure already exists. When taking such an approach, it is critical to have high confidence in the accuracy of any computationally generated structures as RESISTOR is very sensitive to variation in structural input.

Video S6. Demonstration of how to ensure a consistent numbering scheme is used among the structure and sequence file inputs, related to obtaining the sequence and structure files steps 6 and 7

## Key resources table

**Table:** 

REAGENT or RESOURCE	SOURCE	IDENTIFIER
**Deposited data**		

Model of ERK2:AMP-PNP protein structure	Garai et al., 2012^13^	(PDB: 2y9q)
Model of ERK2:SCH7 protein complex structure	Chaikuad et al., 2014^12^	(PDB: 4qta)
cDNA of ERK2	Tate et al., 2019^14^	ENST00000215832.10

**Software and algorithms**		

OSPREY 3.3	Hallen et al., 2018^10^	https://github.com/donaldlab/OSPREY3/releases/3.3-resistor
AmberTools22	Case et al., 2022^25^	http://ambermd.org/GetAmber.php
Maestro	Schrödinger, LLC	https://www.schrodinger.com/products/maestro
Miniconda	Anaconda, Inc.	https://docs.conda.io/en/latest/miniconda.html
Yamllint	Vergé, 2023^26^	https://anaconda.org/conda-forge/yamllint; Vergé A. Yamllint - A Linter for YAML Files. 2023.

## Step-by-step method details

Here we describe the step-by-step details of how to use RESISTOR. These steps include how to 1) specify the K∗ positive and negative designs; 2) run OSPREY to compute each mutant’s positive and negative K∗ scores; 3) process the data to assign mutational probabilities and hotspot scores; and, 4) assign Pareto ranks to each prospective mutant. As a demonstration case, we use RESISTOR to predict ERK2 mutants likely to arise in melanoma that may ablate the efficacy of the ERK1/2 inhibitor SCH7.

### Specifying the K∗ positive and negative designs

**Timing: 1 h**

In this step, we create the YAML files that are used to specify the input for the positive and negative K∗ designs. Positive design refers to improving the interaction between a protein and its endogenous ligand, which in this context is ERK2 with ATP. Negative design refers to ablating the binding between a protein and its targeting inhibitor, here ERK2 and SCH7. By this point, we assume you have completed the steps in the section before you begin, including having downloaded the PDB structure files *4qta.pdb* and *2y9q.pdb*, and the FASTA-formatted cDNA sequence file *ENST00000215832.10.fasta*.

1. Prepare each of the structure files.
  a. Open a terminal shell and activate the AmberTools environment you created in before you begin:
    > conda activate AmberTools22
  b. Run *pdb4amber* on the two ERK2 structures to add any missing atoms:
    > pdb4amber --add-missing-atoms -i 2y9q.pdb -o 2y9q.p4a.pdb
    > pdb4amber --add-missing-atoms -i 4qta.pdb -o 4qta.p4a.pdb
    ***Note:****pdb4amber* renumbers the residues in the input structures, starting from 1. We would like to keep our canonical residue numbering, and luckily *pdb4amber* outputs a mapping file from the original numbers to the new numbers it assigned the residues. This file is titled the name of the input file for *pdb4amber*, suffixed with *_renum.txt*, e.g., 2y9q.p4a_renum.txt. Within the OSPREY distribution there’s a program called *p4a-undo.py* (found in the osprey3.3/resistor directory) which re-assigns the original numbering and chain identifiers.
  c. Using the same AmberTools22 conda environment, run p4a-undo.py with each of the two output structures from *pdb4amber*:
    > python p4a-undo.py 2y9q.p4a.pdb 2y9q.p4a_renum.txt > 2y9q.renum.pdb
    > python p4a-undo.py 4qta.p4a.pdb 4qta.p4a_renum.txt > 4qta.renum.pdb
  d. Add hydrogens to the AMP-PNP and SCH7 structures using a molecular modeling program such as Maestro.
    ***Note:*** Epik^27^ in Maestro^19^ is quite good at correctly predicting $\text{p}K_{a}$ and protonation states for small molecules. You will also need to compute the net charge of the small molecules for step 3, which Epik and Maestro provide. SCH7’s net charge is +1, whereas AMP-PNP has a net charge of -4.
  e. Save the resulting protonated structures as *2y9q.h.pdb* and *4qta.h.pdb.*
    **CRITICAL:** Ensure when saving these protonated structures that the resulting PDB files do not contain trailing whitespace (if it does, remove it using your text editor) and the chain identifiers have been correctly preserved.

2. Split the structure files into their protein and ligand components.
  a. Open *2y9q.h.pdb* in a text editor.
  b. Extract the ATOM records corresponding to ERK2 and save them to a file called *2y9q.erk2.pdb.*
  c. Extract the ATOM records corresponding to AMP-PNP and save them as a file called *2y9q.amppnp.pdb.*
  d. Do the same for *4qta.h.pdb*, saving the corresponding files as *4qta.erk2.pdb* and *4qta.sch7.pdb.*
3. Generate the forcefield parameters and connectivity templates for SCH7 and AMP-PNP.
  a. Activate your AmberTools environment, as in step 1a.
  b. Use the *antechamber* program from AmberTools to generate template files (files with a .*prepi* extension), and *parmchk2* to generate forcefield modification files (files with a *.frcmod* extension):

> antechamber -i 2y9q.amppnp.pdb -fi pdb -o amppnp.prepi -fo prepi -c bcc -nc -4

> parmkch2 -i amppnp.prepi -f prepi -a Y -o amppnp.frcmod

> antechamber -i 4qta.sch7.pdb -fi pdb -o sch7.prepi -fo prepi -c bcc -nc +1

> parmkch2 -i sch7.prepi -f prepi -a Y -o sch7.frcmod

***Note:*** For more information about these and other possible flags to the antechamber and parmchk2 programs, see section 16.1 of the Amber 22 Reference Manual, available from http://www.ambermd.org.^25^

**Figure 2:**
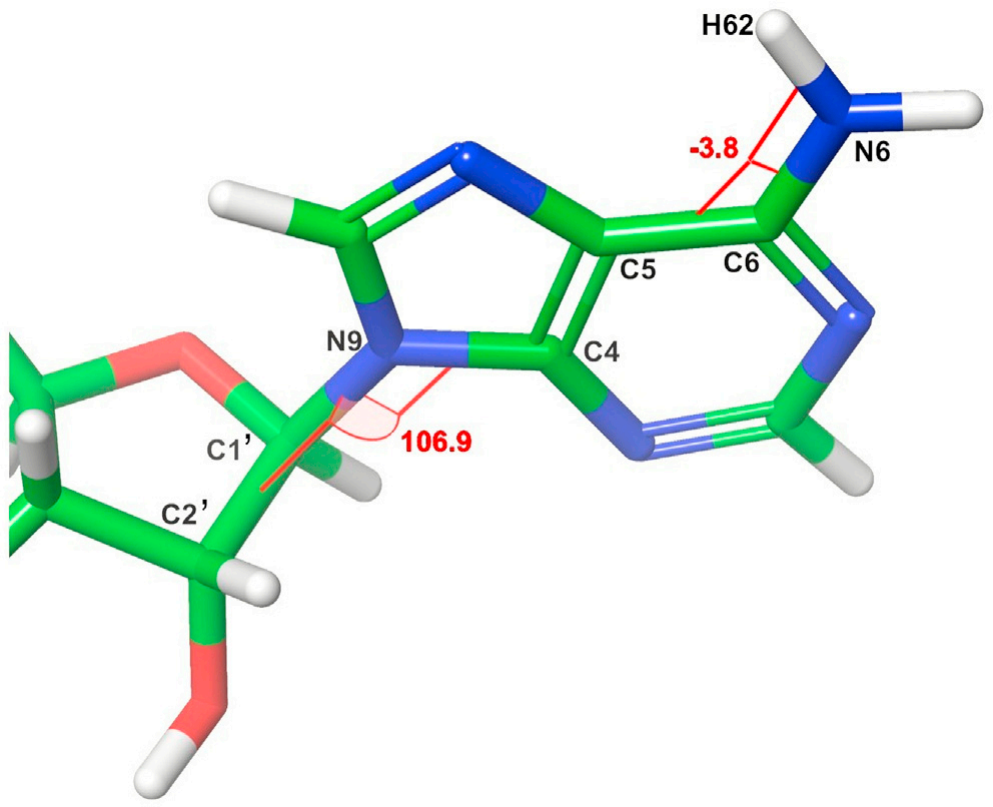
Demonstration of using Maestro to compute the H62-N6-C6-C5 and C2′-C1′-N9-C4 dihedral angles for the extra rotamers definition of AMP-PNP The red lines and numbers show the dihedrals and the computed angles. In Figure 3, these dihedrals are included in the rotamer definition for AMP-PNP. The values -3.8 and 106.9 are rounded to their nearest whole value, -4 and 107, respectively.
  c. Create a text file listing the dihedrals. The format of the file, and the rotamer specification for AMP-PNP is shown in Figure 3.

4. Create template coordinates for the small molecules.
  a. Locate the *gen-templ-coords.sh* script you will use to generate the template coordinates (in the osprey3.3/resistor directory of the OSPREY distribution).
  b. Add the executable bit to the script by running the following command:
    > chmod u+x gen-templ-coords.sh
  c. Run *gen-templ-coords.sh* once for each of the ligands, using Unix pipe redirection to save the output. *gen-templ-coords.sh* expects as input the path of the ligand structure and the three-letter residue name of the ligand used in the structure:
    > ./gen-templ-coords.sh 2y9q.amppnp.pdb ANP > amppnp.tc
    > ./gen-templ-coords.sh 4qta.sch7.pdb 38Z > sch7.tc

5. Generate rotamers for AMP-PNP and SCH7
  a. To allow the ligands to translate, rotate, and flex slightly, we define the flexible dihedrals for the ligands.
  b. Determine the molecule-specific dihedrals using Maestro or other molecular visualization software. Figure 2 demonstrates determining the dihedrals in Maestro.

**Figure 3:**
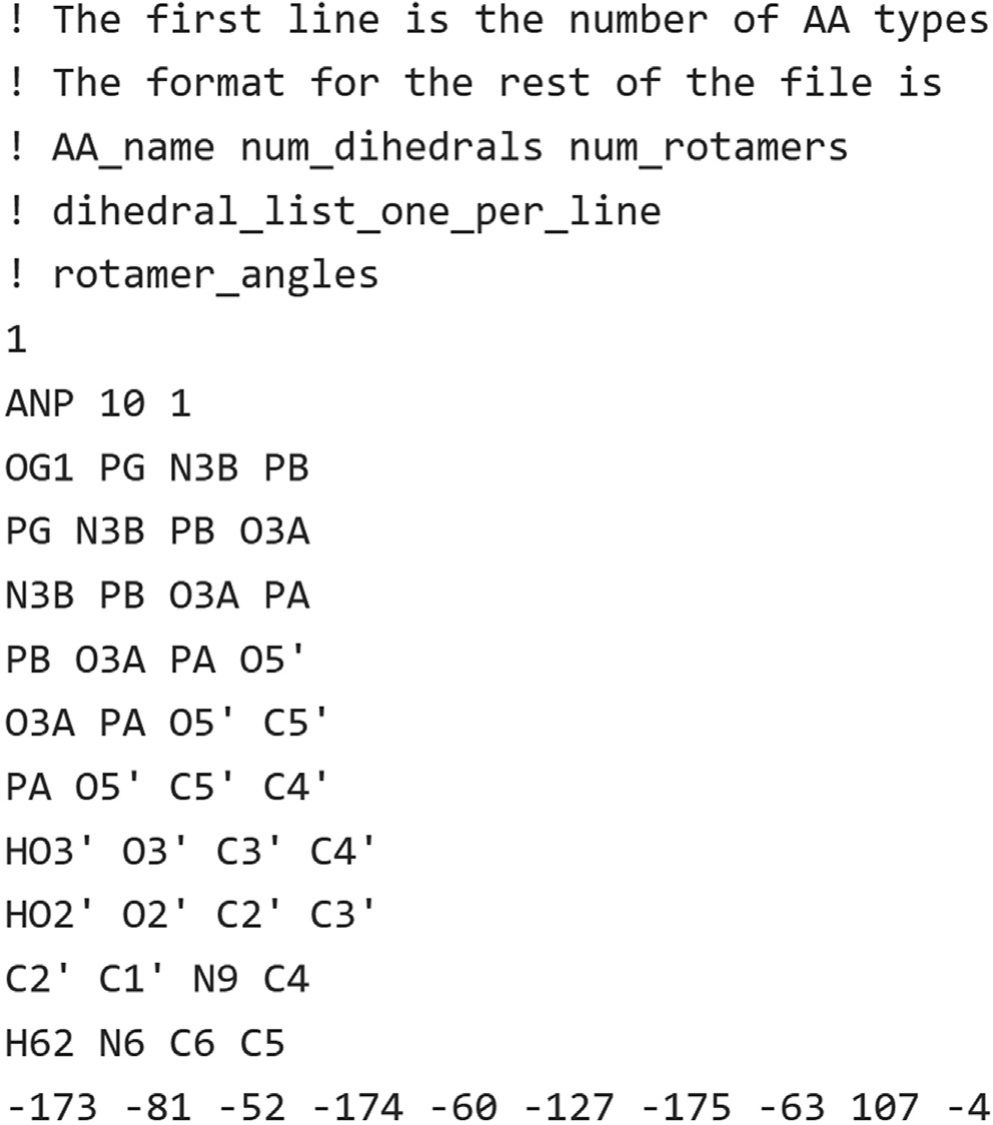
Definition of a rotamer for AMP-PNP. We specify 10 dihedrals These dihedrals allow K∗ in OSPREY to minimize continuously in a voxel around the dihedrals to search for low-energy conformations. This rotamer is defined by its atom names from the PDB file, 2y9q. Lines that begin with an exclamation point (!) are comments. The comments here explain the structure of the file.
  d. Save the file as *amppnp.rot.*
  e. Repeat steps a-d for SCH7, saving that file as *sch7.rot.*

**Figure 4:**
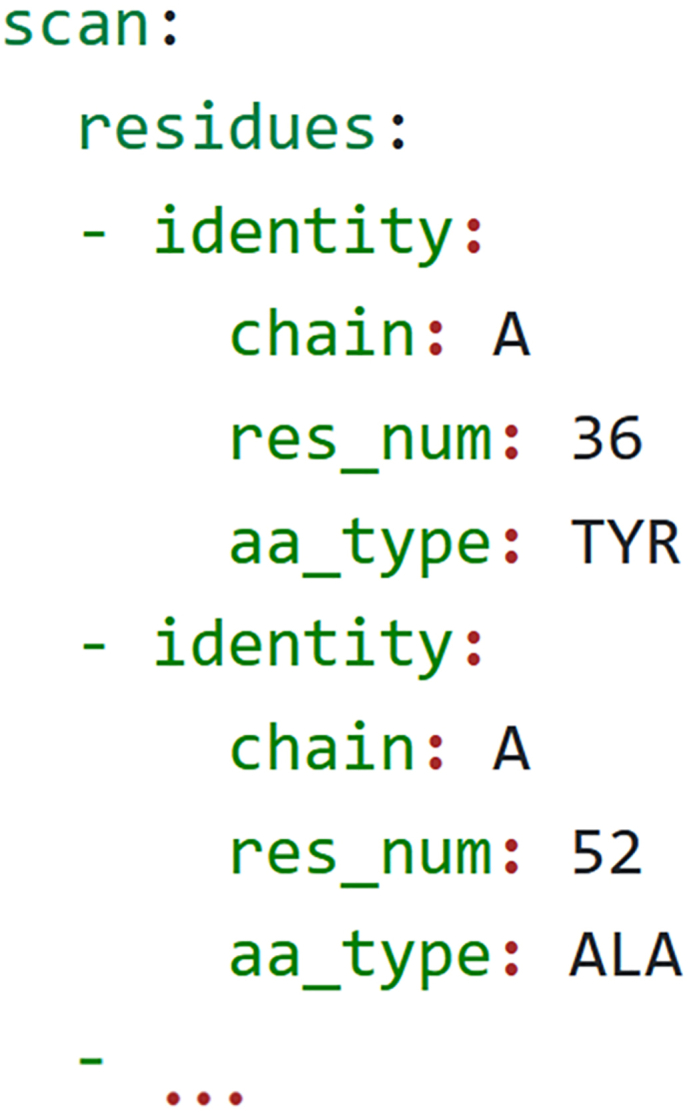
Specification of the scan.residues key The value for the key is a list of objects representing residues in the structure. Each object has a chain key denoting the chain identifier in the structure, a res_num key denoting the residue number, and the aa_type key with the 3-letter amino acid code. In the example above, we specify that Y26 and A52 in chain A of the structure should be included in the scan. Below the ellipsis we would also include objects for I56, R67, E71, Q105, D106, L107, M108, D111, K114, L156, and C166.

**Figure 5:**
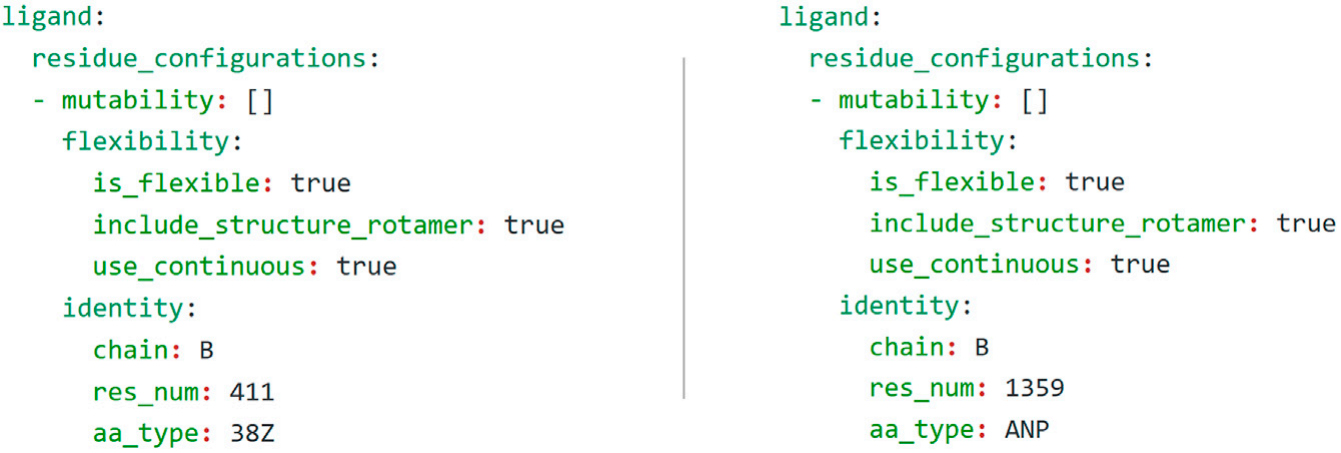
Demonstration of how to specify that the ligand should be flexible in both the positive and negative designs Left: residue 38Z on chain B at position 411 (which is SCH7) is set to be continuously flexible. Right: residue ANP on chain B at position 1359 (which is AMP-PNP) is set to be continuously flexible.

6. Create a template YAML file for the ERK2:AMP-PNP positive K∗ design.
  a. The OSPREY package contains a template K∗ affinity YAML file, located at osprey3.3/resistor/affinity.yaml. Make a copy of this file:
    > cp affinity.yaml erk2-amppnp.yaml
  b. Open the new file in your text editor and incorporate the files you’ve created thus far into the YAML file:
    i. Copy the contents of 2y9q.erk2.pdb as the value for the protein.coordinates key.
    ii. Copy the contents of 2y9q.amppnp.pdb as the value for the ligand.coordinates key.
    iii. Copy the contents of amppnp.tc as the value for the ligand.extra_template_coordinates key.
    iv. Copy the contents of *amppnp.prepi* as the value of the ligand.extra_templates key.
    v. Copy the contents of *amppnp.rot* as the value of the ligand.extra_rotamers key.
      ***Optional:*** You can use a YAML syntax validator, such as yamllint,^26^ to verify you have input syntactically valid YAML.
  c. To verify that you have created the YAML file correctly, run OSPREY to verify the design file:
    > osprey affinity --design erk2-amppnp.yaml --verify-design
    The output of the command should look like:
    WARNING: Using incubator modules: jdk.incubator.foreign
    Design file validated.
    See troubleshooting 2 if your output is different, and troubleshooting 4 if the command output says it can’t parse the YAML file.

7. Create a template YAML file for the ERK2:SCH7 negative K∗ design.
  a. As in step 6a, copy the template K∗ affinity YAML file:
    > cp affinity.yaml erk2-sch7.yaml
  b. Open *erk2-sch7.yaml* in your text editor and incorporate the following files into the negative design specification:
    i. Copy the contents of *4qta.erk2.pdb* as the value for the protein.coordinates key.
    ii. Copy the contents of *4qta.sch7.pdb* as the value for the ligand.coordinates key.
    iii. Copy the contents of *sch7.tc* as the value for the ligand.extra_template_coordinates key.
    iv. Copy the contents of *sch7.prepi* as the value for the ligand.extra_templates key.
    v. Copy the contents of *sch7.rot* as the value for the ligand.extra_rotamers key.
  c. To ensure that your YAML file is in the correct format for OSPREY, use the *affinity* command’s --verify-design flag to check the design file:
    > osprey affinity --design erk2-sch7.yaml --verify-design
    The output of the command should look like:
    WARNING: Using incubator modules: jdk.incubator.foreign
    Design file validated.
    See Video S7 for a demonstration of how to do this step and see troubleshooting 2 if your output is different.

8. Choose residues to mutate and create mutational scan designs.
  a. Taking the files you created in steps 6 and 7, add a YAML list of objects representing these mutants as the value of the scan.residues key, as is shown in Figure 4.
    ***Note:*** For this example, we have chosen to investigate residues Y36, A52, I56, R67, E71, Q105, D106, L107, M108, D111, K114, L156, and C166.
  b. In each of the files generated in steps 6 and 7, set the ligand as flexible by adding it to the ligand.residue_configurations key in the YAML file. Figure 5 shows how this is set in the *erk2-amppnp.yaml* and *erk2-sch7.yaml* files.
  c. After adding these fields, again verify the syntax of the design files is correct:
    > osprey affinity --design erk2-sch7.yaml --verify-design
    > osprey affinity --design erk2-amppnp.yaml --verify-design

9. Generate the K∗ affinity designs for each of the point mutants.
  a. Using the files you modified in step 8, use OSPREY to generate the positive and negative designs for each of the mutants:
    > osprey affinity --design erk2-sch7.yaml --do-scan --scan-flex-distance 2.2
    > osprey affinity --design erk2-amppnp.yaml --do-scan --scan-flex-distance 2.2
    ***Note:*** The --do-scan flag instructs OSPREY to generate a K∗ affinity design centered on each of the residues specified in the scan.residues key. These K∗ affinity designs each include a single mutable residue, which is set to mutate to all the other amino acids, and a flexible shell around the mutating residue. The optional --scan-flex-distance parameter denotes the radius of the OSPREY-generated flexible shell centered on the design's mutable residue. It defaults to 2 Å.
  b. Verify that a positive and negative YAML design specification is created for each of the 13 residues of interest set in step 8a. The naming format of these files is *{original-name}.{residue}.yaml*, e.g., *erk2-sch7.A36.yaml.* There should be a total of 26 newly created files.

Video S7. Demonstration of creating the ERK2:AMP-PNP K∗ affinity YAML design file, related to specifying the K∗ positive and negative designs step 7

### Running the K∗ predictions

**Timing: 1 day to 1 week**

The purpose of this step is to run the positive and negative K∗ mutant predictions with OSPREY. The range in expected time on this step is dependent on how many sequences you’re predicting, the number of flexible residues you’ve configured in your conformation space, and the capabilities of your computer(s). For additional background information on the interpretation of K∗ values and how they are used in predicting resistance mutations, see the *Results* and *STAR Methods* sections of Guerin et al., 2022.^1^

10. Run the positive and negative K∗ designs.
  a. Set the amount of memory to dedicate to the OSPREY process by exporting the JAVA_OPTS environment variable. Set this as high as you can, given the hardware you’re running the design on. Here’s how it could be set on a machine with 760GiB of RAM (while leaving some RAM for the operating system and other processes):
    > export JAVA_OPTS="-Xmx720G -Xms720G"
  b. Execute the *affinity* command in OSPREY on each of the individual mutant design files that you generated in step 9:
    > osprey affinity --design {design-file} --frcmod {frcmod-file}
    where *{design-file}* is the path to one of the design files you generated in step 9, and *{frcmod-file}* is the path to the ligand-specific forcefield modification file you generated in step 3b, e.g.,:
    > osprey affinity --design erk2-sch7.A36.yaml --frcmod sch7.frcmod
    ***Note:*** There are optional flags you can pass to the affinity command that could be helpful for your predictions. These flags include --save-confs, --ensemble-dir, and --cuda . --save-confs takes an integer argument and denotes the number of low-energy conformations from the K∗ molecular ensemble that OSPREY should save of each sequence. It defaults to not outputting structures; if you want structures add this argument and specify a number greater than 0. --ensemble-dir takes a path as an argument and indicates where structures should be saved. And if you have access to CUDA-enabled Nvidia GPUs, you may find that the --cuda flag substantially decreases the amount of time needed to run your designs.
  c. Execute the following command to print the per-residue type K∗ predictions to the terminal screen. If you also want to save the output (both standard out and standard error) to files, you can use Unix pipes to pipe the output to the *tee* program:
    > osprey affinity --design erk2-sch7.A36.yaml --frcmod sch7.frcmod > >(tee -a sch7.A36.stdout) 2> >(tee -a sch7.A36.stderr >&2)
    ***Note:*** There is an optional parameter, --epsilon, which takes a double value as an argument and defaults to 0.683 (see the supplemental information of Ojewole et al. for justification for this default).^2^
    ***Note:***--epsilon must be between 0 and 1; values closer to 0 indicate a more accurate partition calculation and are thus more computationally expensive, and vice-versa. We recommend initially running K∗ affinity designs with an epsilon close to 1, such as 0.9999, and then gradually decreasing epsilon to obtain increasingly accurate K∗ scores while still completing in a reasonable amount of time.

### Assign Pareto Ranks

**Timing: 1 h**

The purpose of this step is to compile and annotate the positive and negative K∗ mutant predictions with mutational signature probabilities and hotspot scores. In addition, we run the *resistor* program to compute the cutoff, *c*, from the K∗ predictions, and filter mutants that K∗ predicts not to be resistance mutants, or whose mutational probability is 0, and assign Pareto ranks.

***Note:*** Your positive and negative K∗ predictions from step 10 should be complete prior to beginning this step.

**Figure 6:**
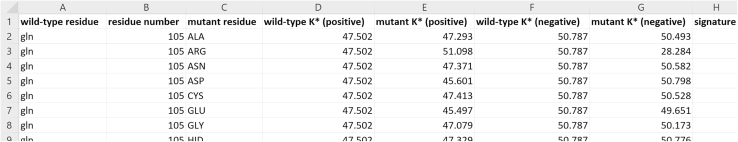
A partially completed worksheet from step 11c The $log_{10}$ K∗ scores for the positive designs (ERK2:AMP-PNP) and negative designs (ERK2:SCH7) are put in columns D-G. Put the K∗ score, for the wild-type sequence, e.g., Q105, bound to the endogenous ligand in column D, and the mutant sequence, e.g., Q105A, bound to the endogenous ligand in column E. In columns F and G do the same for ERK2 bound to SCH7.

**Figure 7:**
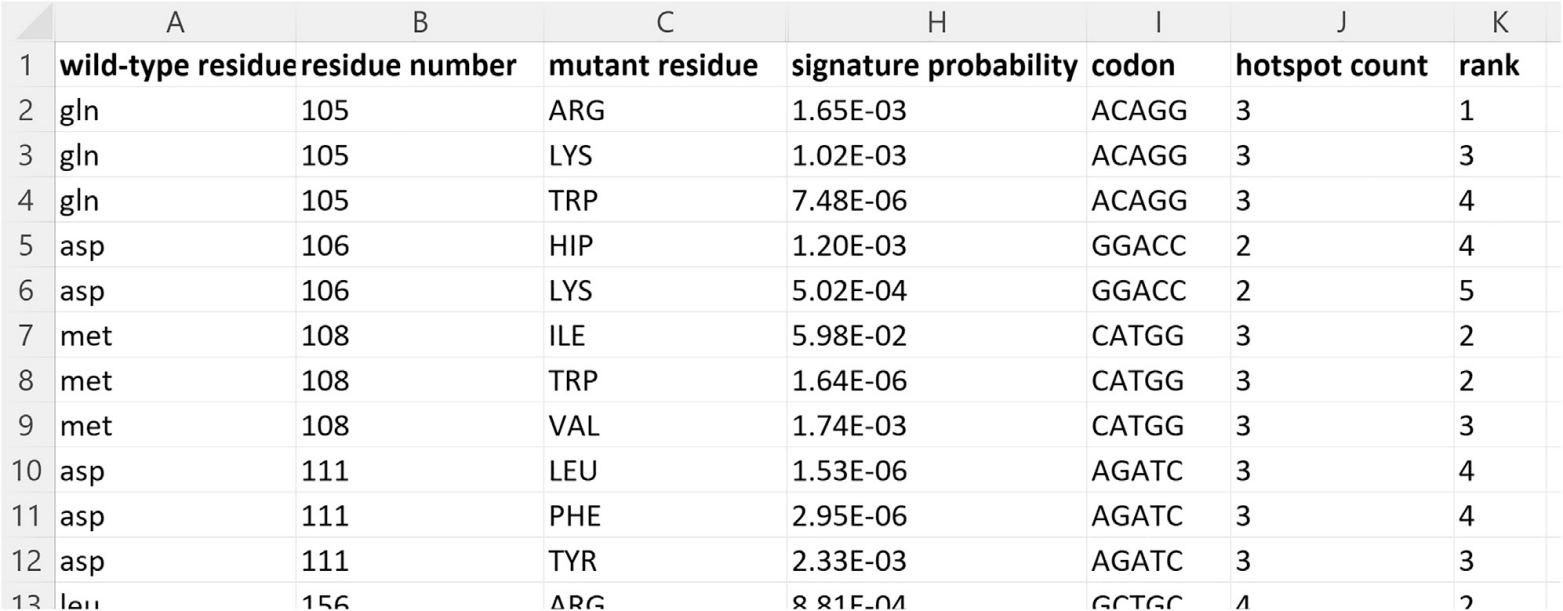
The format of the output file resulting from running step 12c Columns H-K are now filled out. Column H contains the computed signature probability, column I the corresponding codon from the cDNA FASTA file, column J the hotspot count, and column K the computed Pareto rank. The $log_{10}$ K∗ scores for the positive designs (ERK2:AMP-PNP) and negative designs (ERK2:SCH7) are included in columns D-G but are omitted above due to space constraints. The full output file is included in Data S1.

11. Compile the K∗ predictions.
  a. Copy the template CSV file included in the OSPREY distribution (osprey3.3/resistor/resistor.csv) to *erk2-resistor.csv.*
  b. Using the output files from the predictions in step 10, which contain the $log_{10}$ K∗ scores for each of the sequences you evaluated at a particular residue location, fill out the following columns.
    i. *wild-type residue* should have the 3-letter amino acid code for the wild-type residue at *residue number*.
    ii. *residue number* should have the residue number of the residue.
    iii. *mutant residue* should have the 3-letter amino acid code for the mutant residue RESISTOR is evaluating.
    iv. *wild-type K∗ (positive)* should have the $log_{10}$ K∗ score computed on the ERK2:AMP-PNP structure for *residue number*.
    v. *mutant K∗ (positive)* should have the $log_{10}$ K∗ score computed on the ERK2:AMP-PNP structure for *residue number* when *mutant residue* is substituted for *wild-type residue*.
    vi. *wild-type K∗ (negative)* should have the $log_{10}$ K∗ score computed on the ERK2:SCH7 structure for *residue number*.
    vii. *mutant K∗ (positive)* should have the $log_{10}$ K∗ score computed on the ERK2:SCH772894 structure for *residue number* when *mutant residue* is substituted for *wild-type residue*.
  c. Complete a new row for each mutant sequence you evaluated in step 10. Each positive/negative design pair from step 10 evaluated 21 different residue types in each location, meaning we must complete 21 rows for each residue. See Figure 6 for an example of a partially completed worksheet.

12. Run the *resistor* program to assign mutational signature probabilities, filter predicted benign mutations, and assign Pareto ranks.
  a. Open a terminal and change into the osprey3-3/resistor directory.
  b. Download the required Julia dependencies:
    i. Start the Julia interpreter with the following command:
      > julia --project=.
    ii. Activate Julia’s package manager by hitting the ']' key.
    iii. Type instantiate and wait while the package manager downloads the dependencies.
    iv. Exist the interpreter by entering CTRL-D or typing exit() and hitting enter.
  c. Run the program to assign the mutational probabilities and cDNA codons to each mutant sequence:
    > julia --project=. main.jl --mut-prob {mut-prob-file} --fasta {fasta-file} --identifier {id} --csv-file {csv-file} --pareto-config {pareto-config}
    where *{mut-prob-file}* is the path to the mutational probabilities file, *{fasta-file}* is the path to the cDNA file, *{id}* is the identifier of the sequence in *{fasta-file}, {csv-file}* is the path to the CSV file you created in step 11, and *{pareto-config}* is the path to the default Pareto optimization configuration JSON, e.g.,:
    > julia --project=. main.jl --mut-prob osprey3-3/resistor/mutational-signatures/melanoma.json --fasta ./mapk1-cdna.fasta --identifier MAPK1 --csv-file erk2-resistor.csv --pareto-config osprey3-3/resistor/pareto-config.json
    This command.
    i. Fills out the *signature probability* and *codon* columns.
    ii. Filters rows whose *mutant K∗ (positive)* is less than 0, as this indicates the loss of function with the endogenous ligand.^3^
    iii. Filters rows whose *signature probability* is 0 (indicating that the mutant can only occur with 3 base changes).
    iv. Computes the cut-off *c*, as defined in Equation 4 in Guerin et al.^1^
    v. Filters mutants whose ratio of positive to negative K∗ scores is below the cut-off.
    vi. Fills out the *hotspot count* column by counting how many resistance mutations remain at each position after the filtering in the prior steps.
    vii. Fills out the *rank* column by running Pareto optimization over the *mutant K∗ (positive)*, *mutant K∗ (negative)*, *signature probability*, and *hotspot count* columns.
    It outputs the completed table to standard out. You can redirect it to a file using I/O redirection in Linux or by piping the output to the *tee* command. Figure 7 provides an example of the output file.
    ***Note:*** The Pareto JSON specification file is described in the *README.md*. By default, RESISTOR optimizes over mutational signature probability, the positive and negative K∗ scores, and the hotspot score. We've provided a template Pareto JSON specification file in the resistor directory, *pareto-config.json*, which specifies to optimize by maximizing a mutant's signature probability, positive design K∗ score, and hotspot score, and minimizing the mutant's negative design K∗ score. If you had other criteria to optimize over you could add these to this Pareto JSON specification file.
    ***Note:*** There are two additional optional flags to the command above that may be helpful in some circumstances. These flags are --debug and --c0. The --debug flag prints out intermediary CSV files after each filtering and computational step. It also prints the computed cut-off *c* to standard error. The --c0 flag allows you specify a different value for $c_{0}$, for more information as to what this value is see Guerin et al.^1^

## Expected outcomes

RESISTOR provides a protocol for ranking potential resistance mutations to existing and prospective therapeutics. In an earlier publication,^1^ we used RESISTOR to successfully predict resistance mutations in BRAF and EGFR. In this example, we have applied RESISTOR to predicting resistance mutations to SCH7, an ERK1/2 inhibitor.

**Figure 8:**
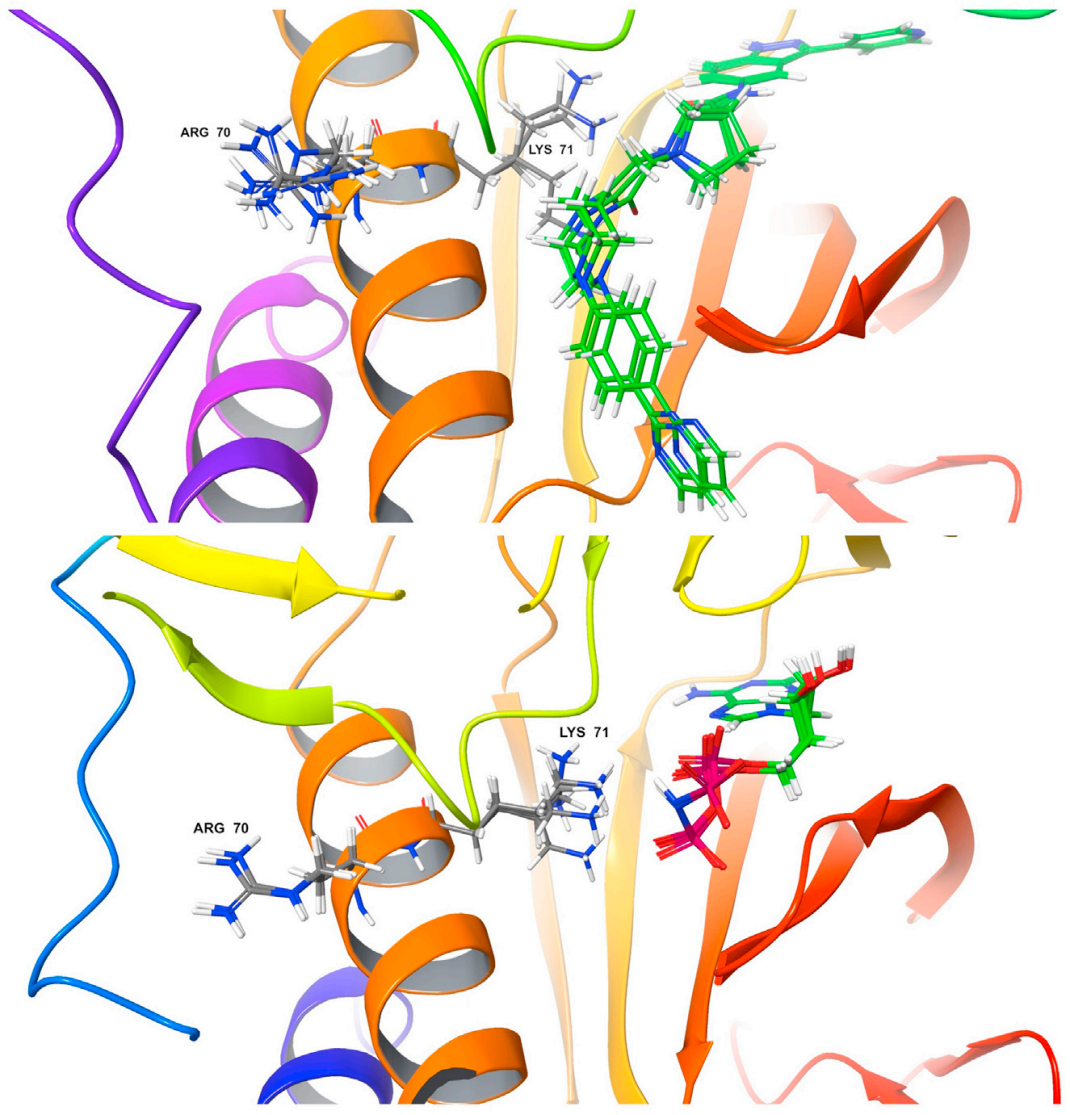
OSPREY-generated structural ensembles of ERK2 E71K Top: ERK2 E71K with SCH7. R70 and 71K are labeled, and SCH7 is in green. Bottom: ERK2 E71K with AMP-PNP. R70 and 71K are labeled, and AMP-PNP is green and purple. According to Brennen et al.,^21^ the E71K mutation grants ERK2 resistance to SCH7. RESISTOR correctly predicts this resistance mutation and ranks it in top Pareto rank. These two OSPREY-generated, low energy structural ensemble files are included in Data S2.

As an outcome, the predicted resistance mutations, as well as their Pareto ranks, are contained in the file output in step 12. With that file, one can analyze the predicted change in a mutant’s positive K∗ score and negative K∗ score, meaning that RESISTOR produces not only binary predictions of a mutant’s resistance or sensitivity profile but also whether a mutation is resistant because of increased binding to the endogenous ligand, decreased binding to the therapeutic, or a combination of the two factors. It also uses a specific cancer type’s mutational signature to predict how likely it is that a putative resistance mutation will occur in a specific cancer patient population. Additionally, as mentioned in step 10, RESISTOR’s use of OSPREY’s K∗ algorithm allows us to output molecular ensembles of low energy conformations for structural analysis. See Figure 8 for an example of the OSPREY-generated low-energy structural ensemble.

## Limitations

In the example we provided above for ERK2 and SCH7, we investigated only potential resistance mutations occurring within the binding pocket of the ligands. Modeling allosteric pathways to resistance, for example mutations distant from the binding pocket on the opposite side of ERK2 causing large-scale conformational rearrangement, while a goal of OSPREY, is not something we’ve yet incorporated into RESISTOR. Additionally, RESISTOR does not model resistance caused by phenomena such as splice variants, amplification, or mutations in related genes, which have been shown to be important in N-RAS, MEK1, MEK2, and other genes.^28^ Additional modeling to incorporate these causes of resistance is left to future work.

## Troubleshooting

### Problem 1

You do not see help text when you run the osprey affinity --help command (related to before you begin, step 5).

### Potential solution

There are different potential causes for this problem. If instead of help text you see the following printed out:

> osprey affinity --help

ERROR: JAVA_HOME is not set and no 'java' command could be found in your PATH.

Please set the JAVA_HOME variable in your environment to match the

location of your Java installation.

then you have not correctly installed and configured Java 17 as detailed in before you begin, step 1. Redo this step and try again. If the message is:

> osprey affinity --help

Error occurred during initialization of boot layer

java.lang.module.FindException: Module jdk.incubator.foreign not found

then it is possible that you are using a version of java that is newer than Java 17. At the current time only Java 17 is supported. It is often the case that there are multiple versions of Java installed in an operating system, and the default version in your operating system may not be Java 17. You can confirm that you are running the correct version of Java for OSPREY by running the command.

$JAVA_HOME/bin/java -version

Below is a demonstration of the output of that command showing the incorrect version of Java:

> $JAVA_HOME/bin/java -version

openjdk version "19" 2022-09-20

OpenJDK Runtime Environment (build 19+36-2238)

OpenJDK 64-Bit Server VM (build 19+36-2238, mixed mode, sharing)

The remedy in this case is to ensure that you have downloaded and configured Java 17, as detailed in before you begin, step 1.

### Problem 2

When using the --verify-design option to the *affinity* command, you see output indicating that indicates a residue was deleted for not having a matching template (related to steps 6 and 7).

### Potential solution

Note which residue the command says it deleted. The output tells you the atoms that it expects to find. Open the YAML file and look at that corresponding residue in either the protein or ligand coordinates. Identify the missing atoms and add them into the structure using a molecular visualization program such as Maestro. If just the labeling is off, fix the labeling. See Video S8 for a demonstration of how to do this.

Video S8. Demonstration of how to address OSPREY affinity deleting a residue for a mismatched template, related to troubleshooting problem 2

### Problem 3

When using the --verify-design option to the *affinity* command, you see output indicating that the residue does not exist (related to steps 6 and 7).

### Potential solution

Look at the coordinates section of the YAML file for the residue mentioned in the error message. Ensure the residue’s number and amino acid identifier matches that used in the scan. See Video S9 for a demonstration of this issue and resolution steps.

Video S9. Demonstration of an error where OSPREY affinity tells you that the residue you specified as flexible does not exist and how to resolve it, related to troubleshooting problem 3

### Problem 4

OSPREY fails to parse the design file YAML specification (related to steps 6 and 7).

### Potential solution

Use a YAML validator, such as yamllint, which can indicate on which line the YAML syntax is broken. Assuming you have installed yamllint as described in step 3 of Installing the Software Dependencies, default invocation of yamllint would look as follows:

> yamllint {design-file}

Any errors will be identified with a description of the problem and the line number. Address them as appropriate. Additionally, the official YAML specification^29^ is a good resource for learning how YAML documents are written and parsed.

### Problem 5

The osprey affinity command begins to run but after some time fail to complete with an error (related to running the k∗ predictions).

### Potential solution

The most common reason osprey affinity fails is that the design has run out of memory. The error output might look like this:

edu.duke.cs.osprey.parallelism.TaskExecutor$TaskException: A task failed, no new tasks can be submitted

at edu.duke.cs.osprey.parallelism.ConcurrentTaskExecutor.recordException(ConcurrentTaskExecutor.java:106)

...

The important thing to remember is that error stack traces are read from the bottom to the top. Scroll to the bottom of the error and if you see a message that looks like:

Caused by: java.lang.OutOfMemoryError: Map failed

at java.base/sun.nio.ch.FileChannelImpl.map0(Native Method)

at java.base/sun.nio.ch.FileChannelImpl.mapInternal(FileChannelImpl.java:1100)

... 18 more

The affinity command failed because it ran out of memory. There are two potential solutions to try. The first is to increase the amount of memory allocated to OSPREY, if possible. This is defined in the JAVA_OPTS environment variable, e.g., to allocate 720 gigabytes to the Java heap, use:

export JAVA_OPTS="-Xmx720G -Xms720G"

For designs where you run out of memory, the first attempt should be to try to make more memory available to OSPREY. If that is not possible, then the second potential solution is to reduce the number of flexible residues in your design. Oftentimes removing one or two flexible residues will allow a previously difficult design to finish. This should only be done when necessary, as removing flexible residue can reduce the accuracy of the predictions.

## Resource availability

### Lead contact

Further information and requests for resources and reagents should be directed to and will be fulfilled by the lead contact, Bruce R. Donald (brd+sp22@cs.duke.edu).

### Materials availability

This study did not generate new unique reagents.

### Data and code availability

The code and datasets used in this study is available at https://github.com/donaldlab/OSPREY3/releases/3.3-resistor (https://doi.org/10.5281/zenodo.7633931), at the Protein Databank (https://www.rcsb.org/structure/2Y9Q and https://www.rcsb.org/structure/4QTA), and the COSMIC database (https://cancer.sanger.ac.uk/cosmic/gene/analysis?ln=MAPK1).

## Supplemental information

Data S1. The Pareto ranked RESISTOR predictions for ERK2 with SCH7, related to assigning the Pareto Ranks, step 12 and Figure 7

Data S2. OSPREY-generated low energy structural ensembles for ERK2 E71K in complex with AMP-PNP and SCH7, related to expected outcomes and Figure 8
